# An Atypical Soft Tissue Response to Maxillary Skeletal Expander: A Case Report

**DOI:** 10.7759/cureus.110148

**Published:** 2026-06-02

**Authors:** Chelsia G Kulathinal, Praveen S Nair, Latheef V P, Sreehari Sathyanathan, Baby Jisha

**Affiliations:** 1 Orthodontics and Dentofacial Orthopaedics, Government Dental College, Kozhikode, Kozhikode, IND

**Keywords:** complication, local inflammation, marpe, oxidative stress, soft tissue proliferation

## Abstract

Mini-implant-assisted rapid palatal expansion (MARPE) is widely used to correct transverse maxillary deficiency in late adolescents and young adults because of its enhanced skeletal effects and minimal dentoalveolar side effects. Although several appliance- and patient-related complications have been reported, unusual soft-tissue reactions remain poorly documented. This case report brings attention to the occurrence of soft tissue proliferation around MARPE, an issue that has received limited emphasis in the literature.

## Introduction

Transverse maxillary deficiency, characterized by a narrow maxillary arch, commonly presents as a posterior crossbite, inadequate arch space, or airway compromise [1,2]. Orthopedic expansion techniques are effective during active growth; however, in adults, progressive ossification and interdigitation of the midpalatal suture reduce the effectiveness of conventional rapid palatal expansion [3].

Mini-implant-assisted rapid palatal expansion (MARPE) has emerged as an effective alternative to surgically assisted procedures in late adolescents and young adults with transverse maxillary deficiency. Several studies have documented its skeletal effects and provided insights into the clinical complications associated with this technique [4-6].

Clinical challenges associated with skeletal expansion devices include appliance-related failures such as loss of stability, appliance fracture, screw fracture, and accidental swallowing or aspiration of appliance components [4,5]. Patient-related complications include periodontal problems involving anchor teeth, endodontic problems, tooth loss, tooth fracture, and asymmetric expansions [6,7].

Although MARPE is considered a safe and effective treatment modality, soft tissue complications have been reported. The biological response begins with local inflammation and tissue remodeling around the implant interface, which may be exacerbated by plaque accumulation, mechanical irritation, excessive activation, or inadequate clearance between the appliance and palatal mucosa [8]. Reported soft tissue complications include palatal mucosal hypertrophy, ulceration [9], and localized infection. Histologic studies have demonstrated more pronounced inflammatory and hyperplastic reactions around mini-implant-supported expanders compared with conventional tooth-borne expanders [10], highlighting the importance of monitoring peri-implant soft tissue healing during treatment.

Complicated sequelae include sinus infections, temporary hearing loss, vomer fracture, papillary loss, nasal bleeding, orbital fracture with partial or temporary sight issues resulting in double vision, cranial-base fracture involving leakage of cerebrospinal fluid [11], unaesthetically widening of the nasal base, and even tremors with tongue irritation [6]. The present case report describes severe soft tissue proliferation associated with a skeletal maxillary expansion device, a clinically relevant complication that has been infrequently discussed in the literature.

## Case presentation

A 19-year-old female patient presented with the chief complaint of difficulty in chewing on the right side and uneven bite and spacing in the upper anterior region. Her general health status was satisfactory, with no significant medical history except for a known allergy to non-noble metal jewelry. To exclude the possibility of an allergic reaction to the appliance material, a patch test was performed and revealed no hypersensitivity to medical-grade stainless steel or nickel. The patch test report is shown in Table 1.

**Table 1:** Patch test report The patient was diagnosed with skeletal maxillary constriction, a prognathic mandible with a vertical growth pattern predominantly contributed by the mandibular component, and facial asymmetry secondary to left-sided hemimandibular elongation as depicted in Figures 1A-1C.

ALLERGEN	RESULT
Nickel Sulphate	Negative
Cobalt Sulphate	Negative
Titanium	Negative
Chromium	Negative
Molybdenum	Negative
Beryllium	Negative

**Figure 1:**
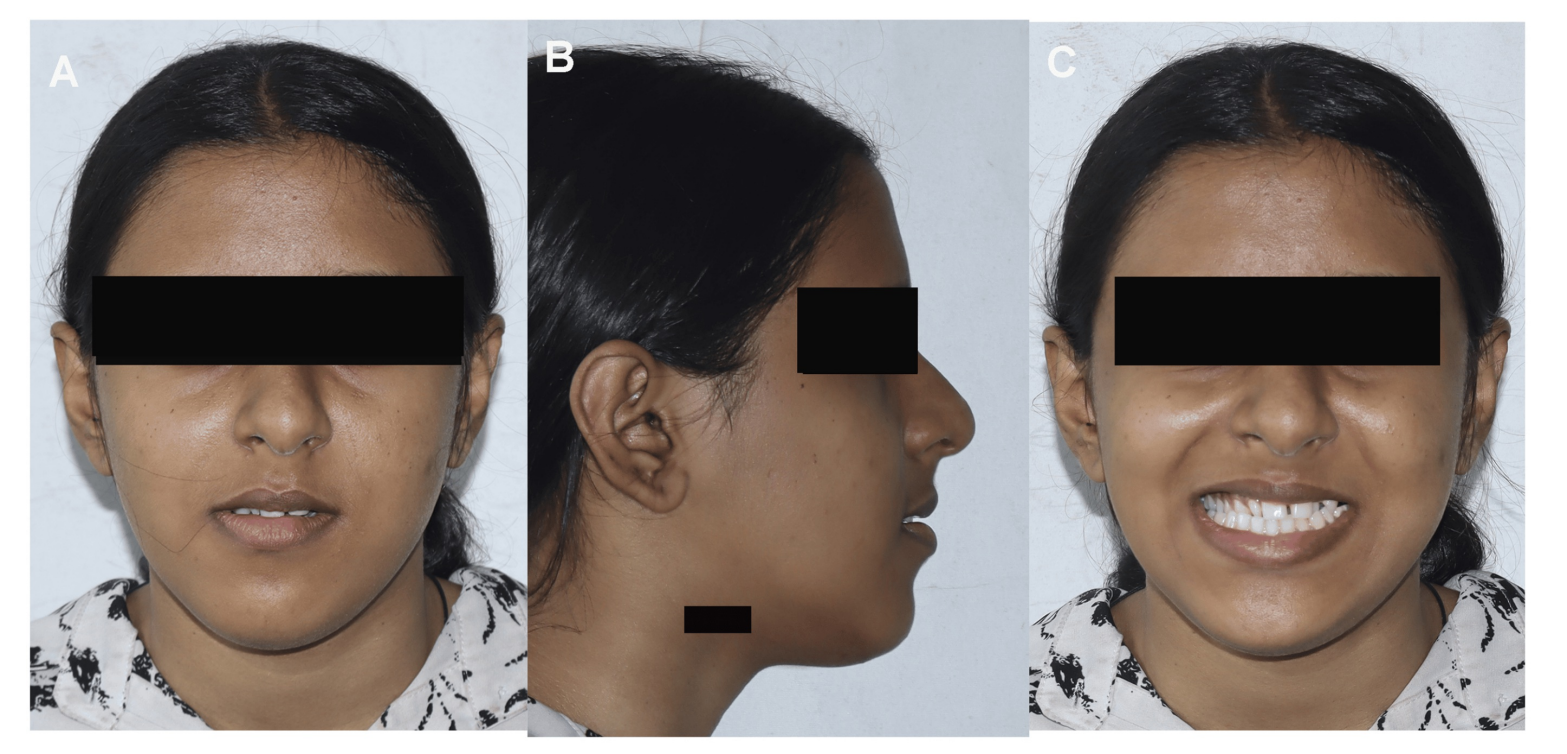
Pre-treatment extraoral photographs A) Frontal at rest B) Profile at rest C) Frontal smiling

The patient was diagnosed with skeletal maxillary constriction, prognathic mandible with a vertical growth pattern predominantly contributed by the mandibular component, and facial asymmetry secondary to left-sided hemimandibular elongation as depicted in Figures 1A-1C.

**Figure 2:**
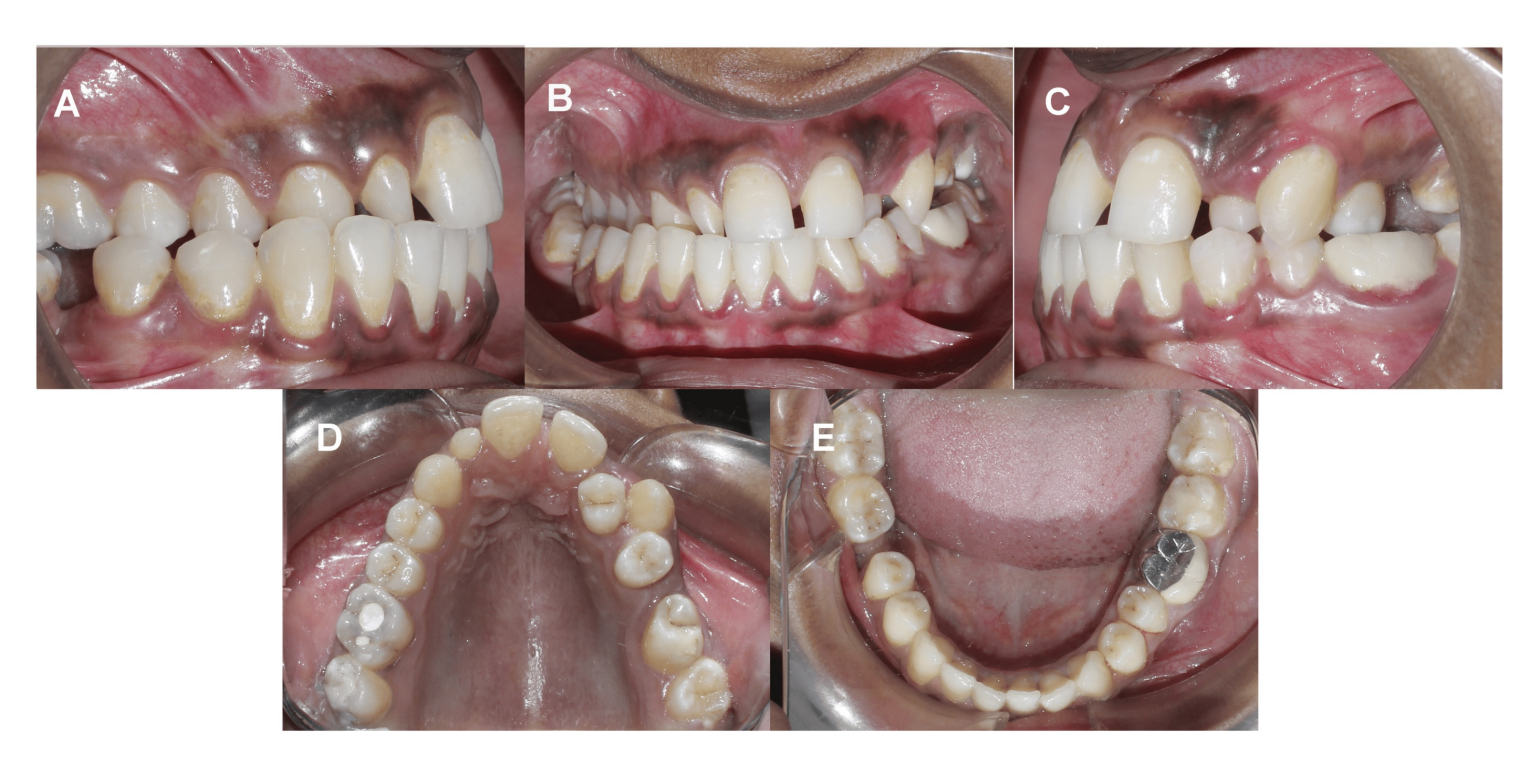
Pre-treatment intraoral images A) Right buccal view B) Frontal view C) Left buccal view D) Maxillary occlusal view E) Mandibular occlusal view

An intraoral examination revealed a mutilated occlusion with missing teeth 22, 26, and 36. Minimal overjet and overbite were present, along with crossbite involving teeth 18 to 12, 22, 24, 25, 27, and 28. Additional findings include transposition of teeth 23 and 24, peg-shaped 12, restoration in 16, and root canal-treated 36 (Figures 2A-2E). The upper dental midline was shifted to the left side by 2 mm, and the lower dental midline was shifted to the right side by 2 mm with respect to the facial midline.

**Figure 3:**
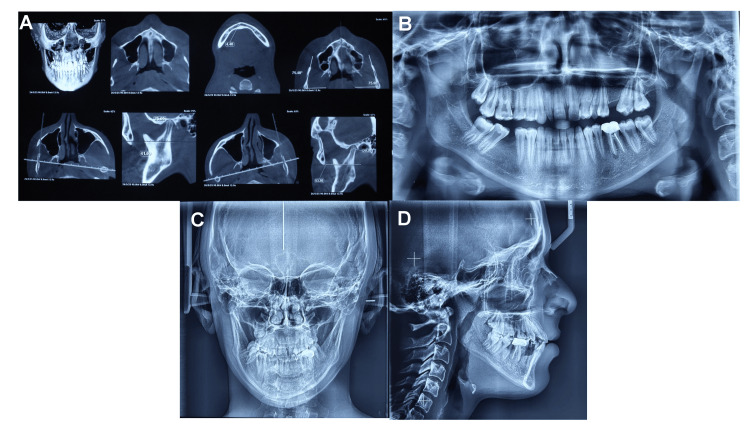
Pre-treatment radiographs A) CBCT B) Panoramic view C) PA cephalogram D) Lateral cephalogram

A panoramic radiograph (OPG), a lateral cephalogram, and posteroanterior (PA) cephalograms (Figures 3B-3D) were assessed. CBCT and model analyses demonstrated an increased buccal corridor and reduced intermolar width of 31 mm (Figure 3A). Based on clinical and radiographic findings, the patient was diagnosed with skeletal class III malocclusion with maxillary constriction, mandibular prognathism, and facial asymmetry.

Treatment objective

The primary treatment objective was correction of the posterior crossbite. The midpalatine suture was assessed through CBCT (Figure 3A), and she was categorized as stage C according to the Angelieri classification of suture maturation [3].

Treatment progress

**Figure 4:**
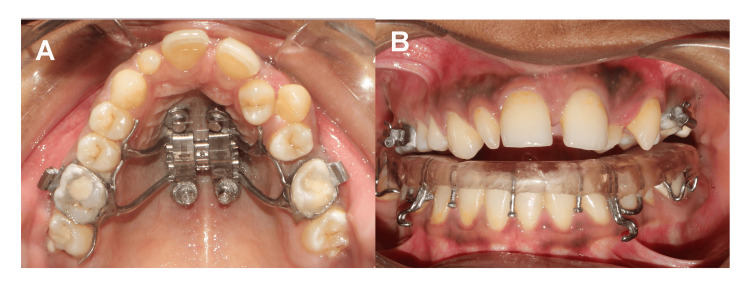
Placement of expander A) Placement of expander B) Placement of mandibular splint

A mini screw-assisted maxillary palatal expander (Leone S.p.A., Florence, Italy), a 6 mm device with first molar bands and anterior wire extensions, was cemented and stabilized by four 2 x 10 mm stainless steel medusa head miniscrews (SK Surgical Industries, India; Grade AISI 316 and FavAnchor™, S.H. Pitkar Ortho Tools Pvt. Ltd., Pune, India) placed in the anterior palate (Figure 4A). A mandibular arch splint was delivered to provide occlusal clearance for crossbite correction (Figure 4B).

**Figure 5:**
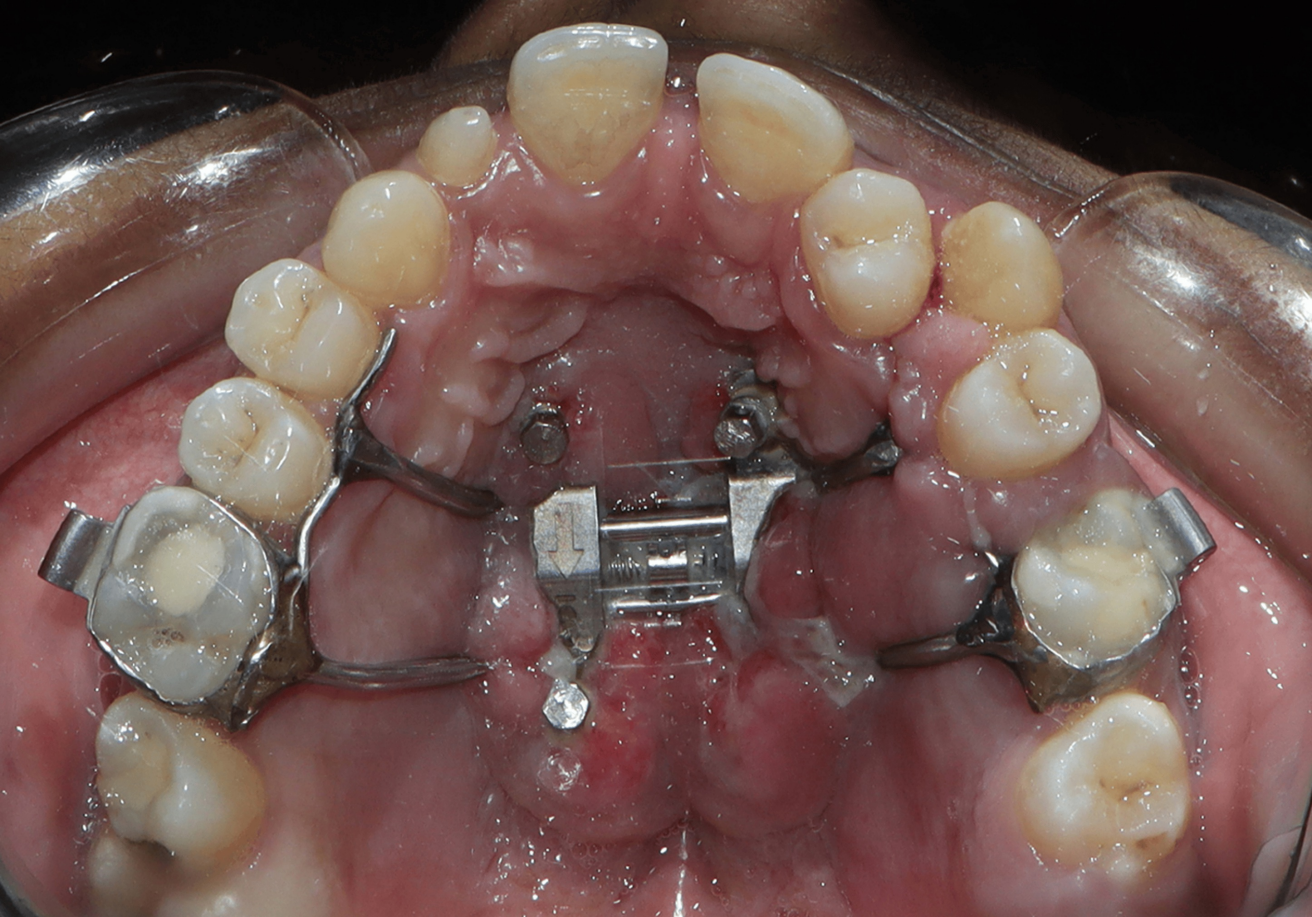
Severe soft tissue inflammation around expander

The patient was prescribed antibiotics and non-steroidal anti-inflammatory drugs and instructed regarding the maintenance of oral hygiene. The activation protocol consisted of 2 turns per day initially, followed by one turn after the appearance of a midline diastema. Two weeks after insertion, the patient reported mild soft tissue inflammation around one of the posterior screws without any additional symptoms. Five weeks after appliance insertion (Figure 5), the patient presented with severe soft tissue inflammation involving posterior screws, skeletal expander, fixation plate, and extension arms on the left side. She also reported a mild burning sensation and discomfort. No other signs and symptoms were present.

Blood investigations demonstrated values within normal limits except for elevated immunoglobulin E (IgE-194.5 IU/mL) levels and reduced hemoglobin levels (10.6 g/dL), indicating anemia as shown in Table 2.

**Table 2:** Laboratory report WBC: White blood cell, RBC: Red blood cell, PCV (HCT): Packed cell volume (Haematocrit), RDW-CV: Red cell distribution width - coefficient of variation, MCV: Mean corpuscular volume, MCH: Mean corpuscular hemoglobin, MCHC: Mean corpuscular hemoglobin concentration, ESR: Erythrocyte sedimentation rate, IU/mL: International units per milliliter

Test	Result	Reference Range
WBC Total Count	6100 cells/cumm	4000 - 11000
RBC	4.50 x10⁶ /µL	3.4 - 5.5
Hemoglobin	10.6 g/dL	12.5 - 15.0
PCV (HCT)	35.9 %	40 - 54
MCV	80.0 fL	80 - 100
MCH	23.5 pg	25 - 35
MCHC	29.4 g/dL	31 - 38
RDW-CV	13.7 %	11 - 16
Platelet Count	3.28 lakhs/cumm	1.5 - 4.5
ESR	15 mm/hour	0 - 20
Immunoglobulin E (IgE)	194.5 IU/Ml *	< 140

**Figure 6:**
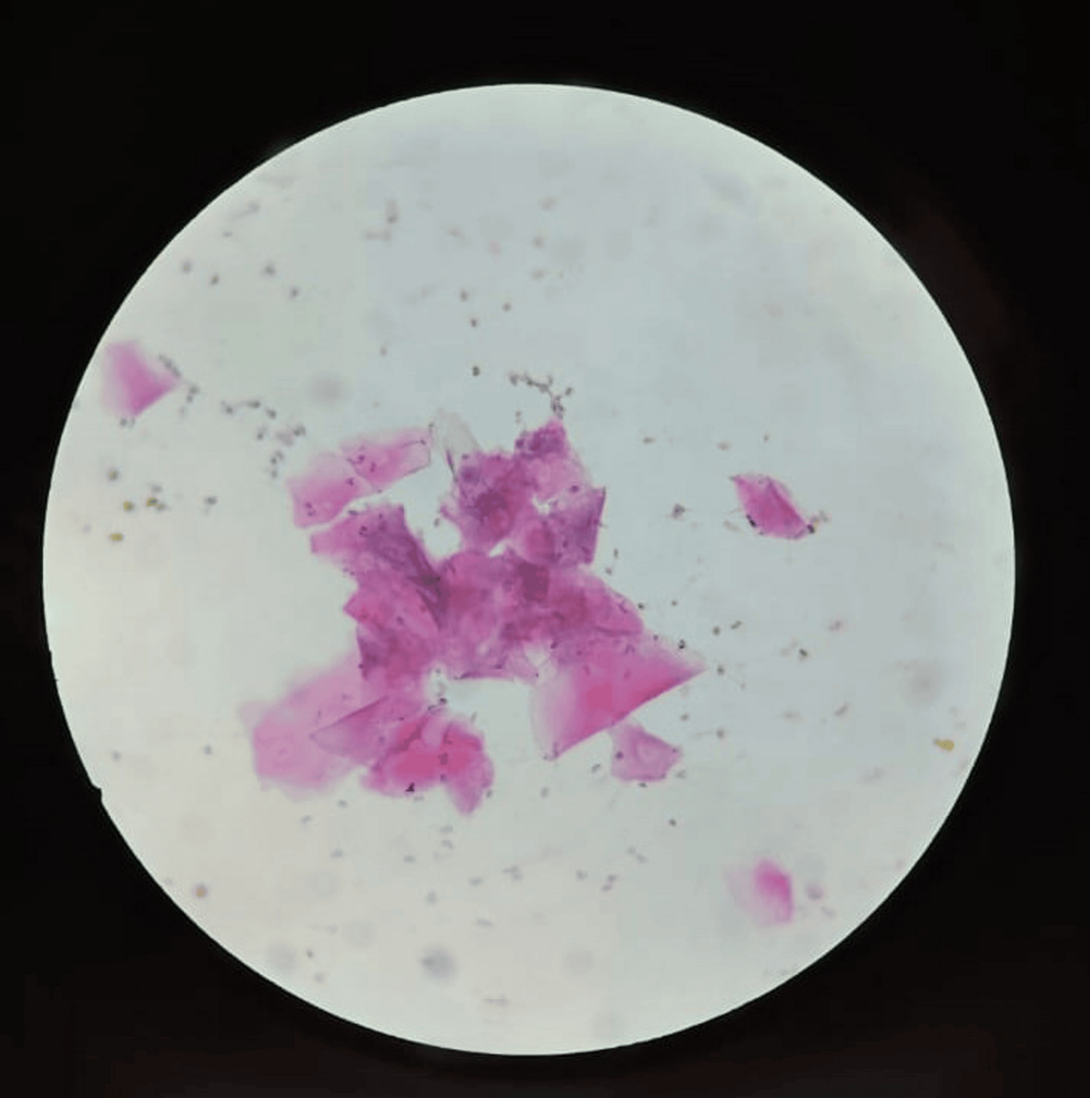
Cytosmear examination

Cytosmear examination revealed only desquamated epithelial cells and inflammatory cells, as shown in Figure 6. 

**Figure 7:**
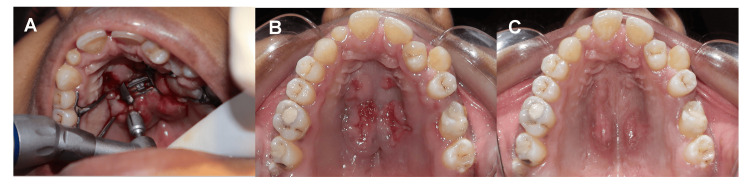
Removal of the expander and healing stages A) Removal of expander B) Healing in 2 days C) Healing in subsequent visits

Ten weeks after insertion, the inflammation had progressed, accompanied by a severe burning sensation and loosening of the posterior screws. The palatal mucosa in the region adjacent to the MARPE appliance appeared erythematous with a predominantly reddish-pink coloration, more pronounced in the mid-palatal area, suggestive of localized inflammatory changes. The affected tissue showed a soft, edematous consistency without evidence of fibrosis or induration. The surface appeared smooth and slightly glossy with loss of normal palatal stippling, consistent with acute inflammatory edema, and no visible ulceration or necrotic changes were observed. The left-side wire extension was associated with localized soft tissue inflammation, whereas no comparable inflammatory changes were observed on the right side. The removal of the appliance and screws was planned under local anesthesia. The posterior screws were embedded within the soft tissue; therefore, surgical incisions were required to expose the screws and facilitate appliance removal, as shown in Figure 7A. A marked reduction in inflammation was observed within two days of appliance removal (Figure 7B), and complete healing was noted during subsequent follow-up visits (Figure 7C). Histopathological evaluation was not performed because the lesion resolved completely following removal of the MARPE appliance and supportive management, eliminating the indication for further invasive investigations.

A clinically significant amount of maxillary expansion was achieved, but not adequate.

## Discussion

For young adults and late adolescents, MARPE offers a reliable option for skeletal maxillary expansion. The benefits include more pronounced skeletal changes, fewer adverse dental effects than standard rapid palatal expansion, minimized periodontal stress, and the elimination of surgical complications [4]. However, the clinical use may be associated with several unfavorable complications, which can be either appliance-related or patient-related. Despite the advantages of the appliance and the limitations noted earlier, this case presented an unforeseen complication characterized by soft tissue proliferation around the bone screws and expansion screws.

Irritation of the surrounding mucosa remains fairly common and can negatively impact treatment if left unmanaged [5]. Major reasons for soft tissue irritation around the implants are either infection of the tissue due to poor oral hygiene or an allergic response to the foreign material. Here, chances of both were ruled out as she was put on antibiotics and a good oral hygiene protocol. Also, her allergic tendency towards medical-grade stainless steel was tested and found to be negative.

The etiology of the complication in this case remains unclear. However, the presence of an elevated IgE level in the absence of a clinically evident allergic reaction may suggest oxidative stress as a possible differential consideration, which is known to activate signaling pathways that promote Th2-mediated immune responses and, consequently, augment IgE-associated inflammation [12,13].

Oxidative stress occurs when reactive oxygen species (ROS) exceed the body’s antioxidant defense (AD) capacity [14]. ROS are chemically reactive molecules that can damage cells and tissues if not neutralized by antioxidants. Appliances can create oxidative stress through two main mechanisms [15] : Metal ion release from brackets and arch wires (nickel, chromium, cobalt, titanium), which can participate in redox reactions (e.g., Fenton reactions) that directly generate ROS. Aseptic inflammation of the periodontal ligament caused by mechanical forces applied to teeth, which triggers inflammatory cytokines, known natural sources of ROS [16].

After appliance insertion, the sudden mechanical loading and potential metal ion exposure together increase ROS production. Since antioxidants do not immediately rise to compensate, the ROS/AD ratio temporarily shifts in favor of ROS, resulting in short-term systemic oxidative stress. By a week or so, endogenous antioxidant systems appear to adapt, restoring balance. However, in some individuals, this physiological adaptation does not occur. This observation highlights the need for further investigation into the potential role of oxidative stress in unusual soft tissue reactions associated with MARPE.

## Conclusions

MARPE is a reliable and effective modality for achieving skeletal maxillary expansion in late adolescents and young adults, offering advantages such as enhanced skeletal effects, reduced dentoalveolar side effects, and avoidance of surgical intervention. Despite these advantages, clinicians should remain aware that unexpected complications beyond commonly recognized appliance or patient-related issues may occur during treatment. This case highlights a rare occurrence of soft tissue proliferation around the mini-implants and expansion screw, where conventional etiological factors such as poor oral hygiene, infection, and material hypersensitivity were ruled out. The absence of these factors, along with the presence of elevated IgE levels, suggests a possible underlying biological mechanism rather than a direct local cause. Although the exact pathogenesis remains uncertain, oxidative stress and the role of reactive oxygen species can be considered differential factors in this atypical tissue response. This case emphasizes the importance of careful monitoring of soft tissue changes during MARPE therapy and highlights the need for further research into the biological pathways involved, as well as the potential role of antioxidant support in managing or preventing such complications.
